# Decision-analytic evaluation of the comparative effectiveness and cost-effectiveness of strategies to prevent breast and ovarian cancer in German women with BRCA-1/2 mutations

**DOI:** 10.1186/s12885-023-10956-6

**Published:** 2023-06-26

**Authors:** Lára R. Hallsson, Gaby Sroczynski, Jutta Engel, Uwe Siebert

**Affiliations:** 1 Institute of Public Health, Medical Decision Making and Health Technology Assessment, Department of Public Health, Health Services Research and Health Technology Assessment, UMIT TIROL - University for Health Sciences and Technology, Eduard-Wallnoefer-Zentrum 1, Hall in Tirol, A-6060 Austria; 2 Division of Health Technology Assessment and Bioinformatics, ONCOTYROL - Center for Personalized Cancer Medicine, Innsbruck, Austria; 3 IBE-Institute for Medical Informatics, Biometry and Epidemiology, LMU-Ludwig-Maximilians- University, Munich, Germany; 4 MCR-Munich Cancer Registry, Institute for Medical Information Processing, Biometry, and Epidemiology, Ludwig-Maximilians Universität (LMU), Munich, Germany; 5 Center for Health Decision Science, Departments of Epidemiology and Health Policy & Management, Harvard T. H. Chan School of Public Health, Boston, MA USA; 6 Institute for Technology Assessment, Department of Radiology, Harvard Medical School, Massachusetts General Hospital, Boston, MA USA

**Keywords:** Ovarian cancer, Breast cancer, Gynecological cancer, Cancer risk, Screening, Prevention

## Abstract

**Background:**

Women with inherited mutations in the *BRCA1* or *BRCA2* genes have increased lifetime risks for developing breast and/or ovarian cancer and may develop these cancers around the age of 30 years. Therefore, prevention of breast and ovarian cancer in these women may need to start relatively early in life. In this study we systematically evaluate the long-term effectiveness and cost effectiveness of different prevention strategies for breast and ovarian cancer in women with *BRCA-1/2* mutation in Germany.

**Methods:**

A decision-analytic Markov model simulating lifetime breast and ovarian cancer development in *BRCA-1/2* carriers was developed. Different strategies including intensified surveillance (IS), prophylactic bilateral mastectomy (PBM), and prophylactic bilateral salpingo-oophorectomy (PBSO) alone or in combination at different ages were evaluated. German clinical, epidemiological, and economic (in 2022 Euro) data were used. Outcomes included cancer incidences, mortality, life years (LYs), quality-adjusted life years (QALYs), and discounted incremental cost-effectiveness ratios (ICER). We adopted the German health-care system perspective and discounted costs and health effects with 3% annually.

**Results:**

All intervention strategies are more effective and less costly than IS alone. Prevention with PBM plus PBSO at age 30 maximizes life expectancy with 6.3 LYs gained, whereas PBM at age 30 with delayed PBSO at age 35 improves quality of life with 11.1 QALYs gained, when compared to IS alone. A further delay of PBSO was associated with lower effectiveness. Both strategies are cost effective with ICERs significantly below 10,000 EUR/LYG or QALY.

**Conclusion:**

Based on our results, PBM at age 30 plus PBSO between age 30 and 40 prolongs life and is cost effective in women with *BRCA-1/2* mutations in Germany. Serial preventive surgeries with delayed PBSO potentially improve quality of life for women. However, delaying PBM and/or PBSO further may lead to increased mortality and reduced QALYs.

**Supplementary Information:**

The online version contains supplementary material available at 10.1186/s12885-023-10956-6.

## Introduction

Women who have inherited mutations in the *BRCA1* or *BRCA2* genes (*BRCA-1/2*) have substantially elevated lifetime risks for developing breast (80–90% lifetime risk) and/or ovarian cancer (18–40% lifetime risk) [1]. *BRCA-1/2* mutation carriers develop breast and ovarian cancer on average 20 years earlier than non-mutation carriers [2].

In Germany, various options for early detection and prevention of breast and ovarian cancer are available for mutation carriers [3]. Currently recommended is intensified surveillance (IS) of the breast, which includes palpation, ultrasound, mammography and magnet resonance imaging (MRI) [3]. Another option is risk-reducing surgery such as prophylactic bilateral mastectomy (PBM) and/or prophylactic bilateral salpingo-oophorectomy (PBSO). PBM is estimated to decrease the risk for developing breast cancer by over 95% [4, 5]. PBSO is estimated to reduce the risk for ovarian cancer by over 90% [6, 7] and the risk for breast cancer by 45% [8].

Despite their potential benefits, all of these options may have negative consequences for the women, and it remains unclear which combination or sequence of preventive interventions at which age may be optimal. In order to make an informed and evidence-based decision on the optimal option, all consequences (i.e., benefits, harms and costs) have to be weighed against each other. Decision-analytic models are commonly used to handle a decision problem of such complex nature using explicit and quantitative methods to identify the optimal options based on utilitarianism [9–11].

Thus, the objective of this study was to develop and apply a decision-analytic model for the evaluation of the long-term effectiveness and cost-effectiveness of different strategies to prevent breast and ovarian cancer in women with *BRCA-1/2* mutations in Germany.

## Methods

### Decision-analytic model

**Fig. 1:**
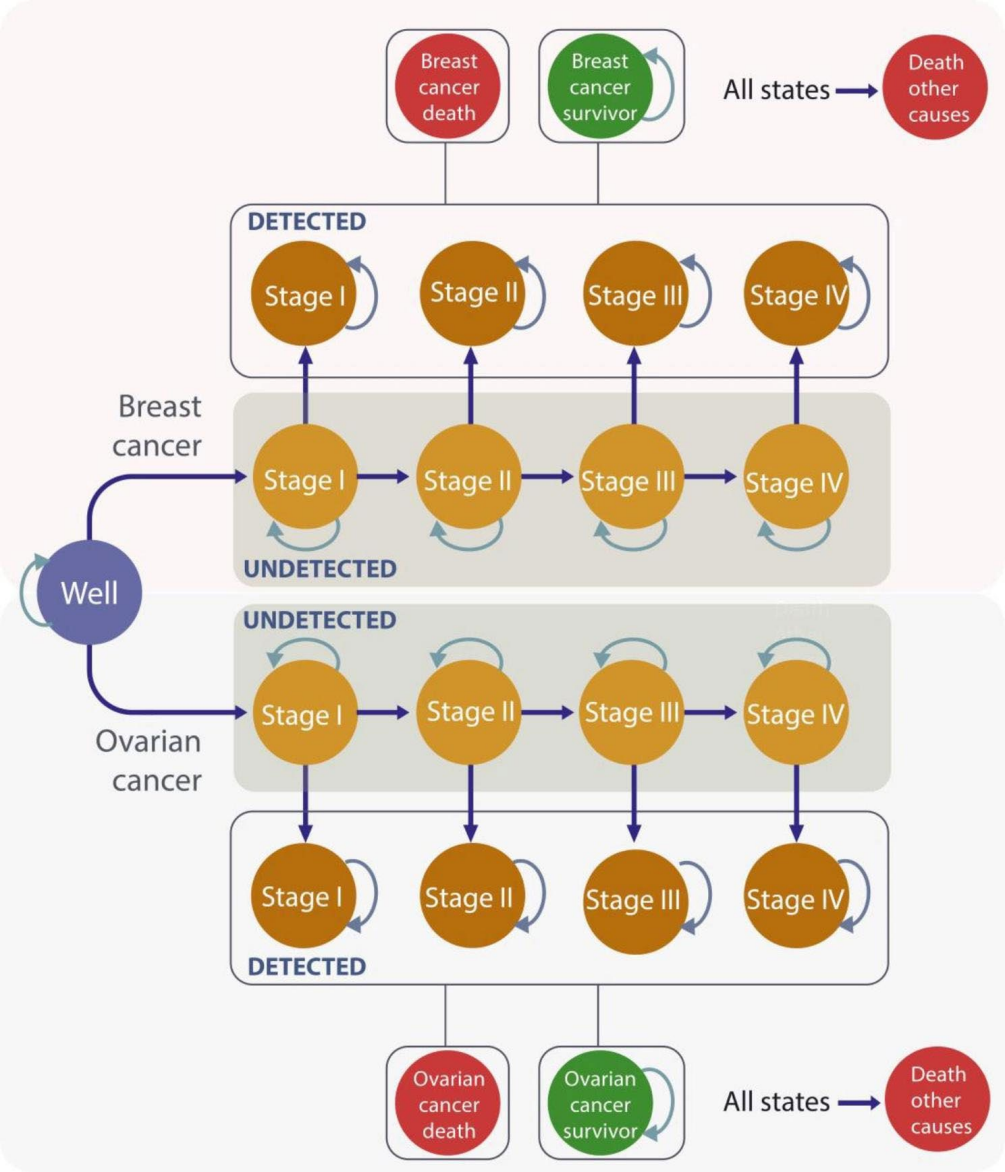
**Illustration of the decision-analytic model.** Health states representing the natural history of breast and ovarian cancer represented in the Markov model are shown as bubbles: No breast and ovarian cancer (well), undetected invasive breast cancer stage I to stage IV, diagnosed invasive breast cancer stage I to stage IV (pT1- pT4), breast cancer survivors 10 years after initial breast cancer diagnosis and treatment (breast cancer survivor), death from breast cancer (breast cancer death), undetected invasive ovarian cancer stage I to stage IV, diagnosed invasive ovarian cancer stage I to stage IV (FIGO states I–IV), ovarian cancer survivors 10 years after ovarian cancer diagnosis and treatment (ovarian cancer survivor), death from ovarian cancer (ovarian cancer death), and death from other causes (death other causes). Progression from one health state to the other is indicated with solid arrows and remaining in the same health state with curved arrows

A decision-analytic Markov state-transition model simulating the natural history of breast and ovarian cancer in women with *BRCA-1/2* gene mutations over a lifelong time horizon was developed (Fig. 1). In a state-transition model a decision problem is conceptualized in terms of a set of (health) states and transitions among these states [12]. A state-transition model was chosen because the natural history of disease can be well described by health states and transitions over time [12]. Since this decision problem can be represented with a manageable number of health states incorporating all characteristics relevant to the decision problem, including the relevant history, a cohort simulation was chosen [12]. In this model, a hypothetical cohort of women moves in annual cycles through different health states over a lifetime (starting at birth) based on stage-specific breast or ovarian cancer occurrence, cancer detection and treatment history (Fig. 1).

Women may remain in the same health state, progress to another health state, may survive cancer or die from cancer or from other causes. We assumed that once detected with cancer, women are treated according to German treatment guidelines [3, 6]. Women initially diagnosed with cancer and treated, may die from ovarian or breast cancer as a function of stage-specific survival rates for each cancer. Women diagnosed with cancer and surviving ten years after initial diagnosis are moving to a cancer survivor health state with a similar mortality risk as the general female population [13]. Recurrent cancer is not modeled explicitly, it is assumed to be included for each cancer state based on survival data linked to initial stage at diagnosis. In all health states women may die from other causes than cancer with age- and gender-specific mortality.

### Compared strategies

Overall, we compared 16 different strategies for breast and ovarian cancer prevention in women with *BRCA-1/2* gene mutations. Strategies were based on current German recommendations [3, 6]. These recommendations state that women with *BRCA-1/2* gene mutations should receive IS for breast cancer as standard care [3] including half-yearly breast palpation plus ultrasound and yearly MRI for the breast plus mammography starting at age 30 [3]. For the detection of ovarian cancer, no IS is recommended [14–16]. The German guideline further suggests offering women PBM as of age 30 [3] and laparoscopic PBSO as of age 40 or when childbearing is completed [6]. We considered the following prevention strategies, which consisted of different single or combined PBM and/or PBSO strategies with different order and age at intervention, and assessed the effects for women who are participating and adhering to these strategies including intensified surveillance: (1) Standard care (IS for breast cancer), (2) PBM at age 30, (3) PBM at age 35, (4) PBM at age 40, (5) PBSO at age 30, (6) PBSO at age 35, (7) PBSO at age 40, (8) PBM plus PBSO at age 30, (9) PBM plus PBSO at age 35, (10) PBM plus PBSO at age 40, (11) PBM at age 30 plus PBSO at age 35, (12) PBM at age 30 plus PBSO at age 40, (13) PBM at age 35 plus PBSO at age 40, (14) PBM at age 35 plus PBSO at age 45, (15) PBM at age 40 plus PBSO at age 45, (16) PBSO at age 35 plus PBM at age 40.

### Model parameters

Annual transition probabilities along with the effectiveness of different strategies used to populate the model are summarized in Table 1. Disease progression parameters of breast and ovarian cancer were calibrated in a systematic and hierarchical fashion to fit age-and *BRCA-1/2*-specific breast and ovarian cancer incidences, derived from literature [2], and stage distribution of breast and ovarian cancer from a German cancer registry (MCR - Munich Cancer Registry) [17, 18]. Details on calibration methods and data used for calibration are summarized in Additional File 1. Stage-specific annual breast and ovarian cancer mortality rates were based on original data from the MCR for the years 1998–2015 [17, 18]. Based on these data, five-year disease specific survival rates for breast cancer stage pT1, pT2, pT3 and pT4 were 99.3%, 87.6%, 59.5%, and 26.2%, respectively [17]; for ovarian cancer FIGO stage I–IV these rates were 87.1%, 70.4%, 35.0%, and 15.5%, respectively [18]. In the model, women could die from causes other than breast and ovarian cancer according to German age-specific all-cause mortality rates for females using German life tables from the German Federal Statistical Office (DESTATIS) [13]. Since all-cause mortality rates from German life tables include females dying of breast or ovarian cancer, background mortality was adjusted for age-specific cancer-related mortality. Age-and cancer-specific mortality rates were derived from the Robert Koch Institute (RKI) [19].

**Table 1:** **Model input data.** Natural history model parameters and effect measures of prophylactic surgeries used to populate the model

*Progression*		*Annual transition probability*		*Reference*
*From*	*To*			
***Age-specific progression from well to undetected breast cancer pT1***				
		Age		
Well	Und. pT1	20–29	0.0002–0.0020	Antoniou et al., 2003 [2]*
		30–39	0.0106–0.0185	
		40–49	0.0326–0.0688	
		50–59	0.0705–0.0723	
		60–69	0.0740–0.0757	
		70–79	0.0666 − 0.0638	
		80–89	0.0609 − 0.0583	
		90–99	0.0557 − 0.0531	
***Age-specific progression from well to undetected ovarian cancer stage FIGO I***				
		Age		
Well	Und. FIGO I	20–29	0.000015-0.00002	Antoniou et al., 2003 [2]*
		30–39	0.0019–0.0025	
		40–49	0.0119–0.0178	
		50–59	0.0242–0.0242	
		60–69	0.0348–0.0433	
		70–79	0.0465–0.0553	
		80–89	0.0497 − 0.0441	
		90–99	0.0385 − 0.0328	
***Progression of undetected cancer to more severe stages***				
***Breast cancer***				§
Und. pT1	Und.pT2	0.205		
Und. pT2	Und. pT3	0.25		
Und. pT3	Und.pT4	0.60		
***Ovarian cancer***				
Und. FIGO I	Und. FIGO II	0.70		
Und. FIGO II	Und. FIGO III	0.93		
Und. FIGO III	Und. FIGO IV	0.99		
***Symptomatic detection: Transition from undetected to detected cancer***				
Und. pT1	Det. pT1	0.23		§
Und. pT2	Det. pT2	0.55		
Und. pT3	Det. pT3	0.60		
Und. pT4	Det. pT4	1.00		
Und. FIGO I	Det. FIGO I	0.15		
Und. FIGO II	Det. FIGO II	0.15		
Und. FIGO III	Det. FIGO III	0.72		
Und. FIGO IV	Det. FIGO IV	0.95		
***Cancer survival and mortality***				
***Disease- and stage-specific survival (year 1–10)*** ^†^				
pT1	Survivor (pT1)	1.0–0.988		MCR, 2017 [17]
pT2	Survivor (pT2)	0.991 − 0.966		MCR, 2017 [17]
pT3	Survivor (pT3)	0.911–0.939		MCR, 2017 [17]
pT4	Survivor (pT4)	0.735–0.874		MCR, 2017 [17]
FIGO I	Survivor (FIGO I)	0.981 − 0.956		MCR, 2017 [18]
FIGO II	Survivor (FIGO II)	0.918–0.988		MCR, 2017 [18]
FIGO III	Survivor (FIGO III)	0.809–0.963		MCR, 2017 [18]
FIGO IV	Survivor (FIGO IV)	0.576–0.989		MCR, 2017 [18]
***Effect measures: Relative risk (surgery vs. no surgery)*** ^‡^				
***Breast cancer***				
PBM		0.070		Rebbeck et al., 2004 [41]
PBSO		0.500		Rebbeck et al., 2002 [7]
PBM + PBSO		0.039		Rebbeck et al., 2004 [41]
***Ovarian cancer***				
PBSO		0.038		Rebbeck et al., 2002 [7]

det.: detected; FIGO I-IV: ovarian cancer stage FIGO 1–4; MCR: Munich Cancer Registry; PBM: Prophylactic bilateral mastectomy; PBSO: Prophylactic bilateral salpingo-oophorectomy; pT1-4: breast cancer stage pT1-pT4; und.: undetected, vs.: versus

* Calibrated to *BRCA-1/2* specific incidence rates reported in Antoniou et al. 2003 [2] (see Additional file 1: Table S1)

§ Progression probabilities for each stage and detection probabilities were assessed through calibration to incidence rates and stage distribution of breast and ovarian cancer respectively

† Annual stage-specific survival probabilities (year 1–10 after diagnosis) were used to populate the model. Here shown as a range

‡ All women are assumed to receive standard care (i.e., intensified surveillance of breast cancer)

We used quality-of-life indices (“utilities”) to weight life years with the specific health-related quality of life women experience in different health states. Utilities range from 0 (i.e., reflecting death) to 1 (reflecting best possible health) [20]. Utilities were derived from literature and included stage-specific utility values for diagnosed cancer patients with *BRCA-1/2* mutations and utility values after prophylactic surgery (Table 2). Utility for undetected cancer was approximated using data from literature (see Additional file 2). We assumed the utility of (healthy) mutation carriers to be reduced due to prophylactic surgery (Table 2) and we assumed the women’s health-related quality of life to increase after prophylactic surgery to regain the utility of the women’s preceding health state in the subsequent year. This was implemented through a relative disutility applied to the base utility of the current health state.

**Table 2:** **Utilities used for different health states in the model.**

*Health status*	*Utility (SD)*	*Reference*
Well at age 30 with *BRCA-1/2* mutation	0.92 (0.15)	Grann et al. 2010 [42]*
***Breast cancer*** ^***¶***^		
pT1	0.68 (0.06)	Schleinitz et al., 2006 [43] ^§^
pT2	0.61 (0.06)	Schleinitz et al., 2006 [43] ^§^
pT3	0.56 (0.06)	Schleinitz et al., 2006 [43] ^§^
pT4/ metastatic	0.42 (0.06)	Schleinitz et al., 2006 [43] ^§^
Clinical remission (breast cancer survivors) ^†^	0.83 (0.24)	Havrilesky et al., 2009 [44]
***Ovarian cancer*** ^***¶***^		
FIGO I	0.81	Havrilesky et al., 2009; Kearns et al., 2016 [44, 45]*
FIGO II	0.72	Havrilesky et al., 2009; Kearns et al., 2016 [44, 45] *
FIGO III	0.63	Havrilesky et al., 2009; Kearns et al., 2016 [44, 45] *
FIGO IV/metastatic	0.55	Havrilesky et al., 2009; Kearns et al., 2016 [44, 45] *
Clinical remission (ovarian cancer survivors)	0.83 (0.25)	Havrilesky et al., 2009 [44]
***Prophylactic surgeries***		
PBM	0.88 (0.24)	Grann et al., 2011 [33]*
PBSO	0.95 (0.24)	Grann et al., 2011 [33]*
PBM + PBSO	0.84 (0.25)	Grann et al., 2011 [33]*

FIGO I-IV: ovarian cancer stage FIGO 1–4; MRI: Magnetic resonance imaging; PBM: Prophylactic bilateral mastectomy; PBSO: Prophylactic bilateral salpingo-oophorectomy; pT1-4: breast cancer stage pT1-pT4

^***¶***^ Utility values for undetected cancer were approximated using utility values for patients without depression or depression-like symptoms. For details see Additional file 2

* Time trade-off (TTO) method to estimate utility

§ Standard Gamble (SG) approach to estimate utility

† Utility for Breast cancer clinical remission is assumed to be the same as for ovarian cancer clinical remission

German direct medical cost data were derived from published literature including costs for IS, surgical procedures, medications, and other treatment procedures [21]. All costs were converted to the target year (2022) using the Gross Domestic Product deflator index (GDPD values) [22]. All women received continued intensified breast cancer surveillance at an average cost of €608 per year [21]. We assumed that women undergoing PBM (with or without PBSO) incur only half of these costs as MRI is excluded from surveillance in these cases. Estimates of prophylactic surgical costs were based on the published literature [21].

For drug costs, we separated non-advanced (FIGO I + II) from advanced (FIGO III + IV) ovarian cancer and non-metastatic (stage pT1-3) from metastatic breast cancer (stage pT4). Overall, proportions of women with non-metastatic breast cancer receiving adjuvant radiotherapy, chemotherapy, and endocrine therapy were based on data from the German Consortium for Hereditary Breast and Ovarian Cancer database derived by Müller et al. 2018 [21]. For women with metastatic breast cancer cost data were obtained from literature [21]. The chemotherapeutic regimens most frequently prescribed in Germany were assumed to be equally distributed among women. Breast cancer drug costs were estimated for specific cancer type subgroups. Both breast and ovarian cancer therapy costs for initial treatment (i.e., surgical, chemotherapy, medication, and other treatment costs) were implemented as one-time costs at cancer diagnosis. Women were assumed to receive follow-up treatment for ten and five years after initial treatment for breast and ovarian cancer, respectively [3, 6]. Annual costs for follow-up treatment include IS as well as treatment of recurrent cancer. Recurrent cancer was assumed to be treated at the same costs as initial cancer treatment. Recurrent cancer was assumed to be included for each cancer state based on survival data linked to initial stage at diagnosis. Costs of palliative care were considered for all women dying from metastatic breast or ovarian cancer. Aggregated one-time initial treatment and palliative care costs as well as aggregated annual follow-up costs (in 2022€) are summarized in Table 3.

**Table 3:** **Aggregated costs (in 2022€) for diagnostic, preventive, and therapeutic procedures.** All Women receive intensified surveillance for breast cancer as standard care. Prophylactic surgeries, initial treatment and palliative care are assumed to occur only once at a one-time cost. Follow-up costs are annual costs over 5 and 10 years after cancer diagnosis for ovarian and breast cancer, respectively. All costs are based on the published literature [21]

*Procedure*	*Costs (in 2022€)*
*Standard care and prophylactic surgeries*	
Standard care (intensified surveillance for breast cancer) *	608
PBM ^§^	9,032
PBSO ^§^	3,099
PBM + PBSO^§^	12,131
***Initial cancer treatment (surgery, drugs, radiotherapy etc.)*** ^§^	
Breast cancer (pT1-pT3) (incl. standard care)	20,092
After PBM	14,333
After PBSO	20,092
After PBM + PBSO	14,333
Breast cancer (metastatic) (incl. standard care)	30,623
After PBM	24,865
After PBSO	30,623
After PBM + PBSO	24,865
Ovarian cancer (non-advanced FIGO I-II) (incl. standard care)	13,666
After PBM	13,666
After PBSO	11,280
After PBM and PBSO	11,280
Ovarian cancer (advanced FIGO III-IV) (incl. standard care)	35,052
After PBM	35,052
After PBSO	32,666
After PBM and PBSO	32,666
***Palliative care costs*** ^§^	
Metastatic/end stage breast cancer	12,103
Metastatic/end stage ovarian cancer	12,103
***Annual follow-up costs***	
Breast cancer (incl. Standard care); 10 years of follow up*	
pT1	595
pT2	652
pT3	589
pT4	686
pT1 after PBM	443
pT2 after PBM	500
pT3 after PBM	437
pT4 after PBM	534
pT1 after PBSO	595
pT2 after PBSO	652
pT3 after PBSO	589
pT4 after PBSO	686
pT1 after PBM and PBSO	443
pT2 after PBM and PBSO	500
pT3 after PBM and PBSO	437
pT4 after PBM and PBSO	534
Ovarian cancer (incl. Standard care); 5 years of follow up*	
Non-advanced (FIGO I + II)	1,356
Advanced (FIGO III + IV)	2,389
Non-advanced (FIGO I + II) after PBM	1,052
Advanced (FIGO III + IV) after PBM	2,085
Non-advanced (FIGO I + II) after PBSO	1,214
Advanced (FIGO III + IV) after PBSO	2,246
Non-advanced (FIGO I + II) after PBM and PBSO	910
Advanced (FIGO III + IV) after PBM and PBSO	1,942

FIGO I-IV: ovarian cancer stage FIGO grade 1–4; incl.: including; PBM: Prophylactic bilateral mastectomy; PBSO: Prophylactic bilateral salpingo-oophorectomy; pT1-4: breast cancer stage grade pT1-pT4

*annual costs

§ one-time costs

### Statistical analyses

#### Base-case analysis

We performed a deterministic cohort simulation over a lifelong time horizon starting the evaluation at 30 years to predict the following outcomes: reduction in breast and ovarian cancer incidence (in %) and cancer-specific mortality (in %), undiscounted life expectancy (in life years (LYs)), undiscounted quality-adjusted life years (QALYs), total lifetime costs, discounted incremental cost-effectiveness ratios (ICER) expressed in Euros (€) per life-year gained (LYG) and discounted incremental cost-utility ratios (ICUR) in Euros (€) per QALY gained. As a point of reference, a willingness-to-pay threshold (WTP) of 90,000 €/LYG or QALY was assumed [23]. We adopted the German health-care system perspective and discounted costs and health effects by 3% annually in the cost-effectiveness analyses [24]. Strategies are considered dominated if they provide less health benefit at higher costs when compared to any other strategy. As dominated strategies should not be considered by decision makers, they were eliminated from the calculation of cost-effectiveness ratios. Furthermore, extended dominance was applied to eliminate strategies, for which costs and benefits are dominated by a mix of two other alternatives [25]. We used an efficiency frontier approach to assess and visualize the trade-off between benefits and costs [24, 26]. This approach excludes strategies that have a smaller benefit and greater cost than any other (combination of) strategy due to dominance. The curve connecting all non-dominated strategies is called the efficiency frontier. Comparisons are made in a stepwise fashion comparing each strategy with the next effective strategy on the efficiency frontier using ICERs and ICURs. The model was programmed in TreeAge Pro Version 2023 [27].

#### Sensitivity analyses

Deterministic one-way sensitivity analyses were performed on utilities, intervention effect measures, costs and the discount rate to estimate the uncertainty surrounding model assumptions and input parameters and to assess the robustness of the results [28]. Utilities and effect measures were varied between a 20% increase and reduction, the discount rate was varied from 1 to 10% and costs were varied between 50% and 200% of the base case value. Percentages in relation to base case value were used for sensitivity analyses as there were no predefined ranges of values for the different model parameters available from literature.

#### Model validation

The model was validated on four levels: [1] technical verification for face validity, [2] internal validation (e.g., debugging, consistency and plausibility checks), [3] cross-model validation and [4] external validation with historical data [29].

All methods were performed in accordance with relevant guidelines which are referred to throughout the methods section.

## Results

### Model validation

Internal validation showed that the model predictions were consistent with epidemiological data used in the model. In cross-model and external validations, the model compared well with other published models and to historical *BRCA-1/2* specific data not used to populate the model. The model-predicted risk for developing breast cancer until the age of 70 years was 68%, and thus comparable to observed data for *BRCA-1/2* mutation carriers provided by the literature ranging between 57% [2] and 84% [30, 31]. The model-predicted ovarian cancer risk until the age of 70 years was 21%, which compares, although slightly lower, still well with observed data from one study [2] with 28% (CI: 11–36%). For both cancer types, the model predicted a risk for developing cancer of 90% until the age of 70 years, which is similar to an estimate of 92% by Easton et al. [30] and Ford et al. [31]

### Clinical effectiveness

All intervention strategies were more effective than IS. Over a lifetime, for the different evaluated prevention strategies, the decision analysis resulted in a relative risk reduction for developing breast cancer between 23.0 and 85.3% and for dying from breast cancer between 25.9 and 86.8% when compared to standard care (i.e., IS for breast cancer). Compared to standard care, the predicted life-time relative risk reduction for ovarian cancer ranged from 8.6 to 81.8% and the predicted reduction in ovarian cancer mortality ranged from 12.9 to 83.3%. For both cancer types, these benefits were highest for women undergoing PBM plus PBSO at age 30.

For the different prevention strategies, the average gain in undiscounted life expectancy and quality-adjusted life expectancy was 2.0–6.3 LY and 3.6–11.1 QALYs, respectively, when compared to standard care.

**Fig. 2:**
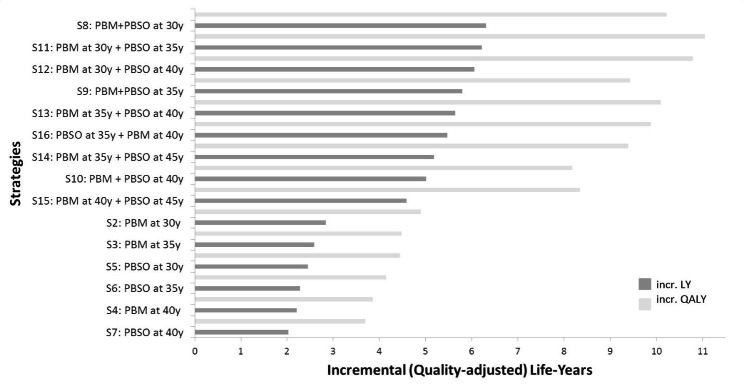
Clinical effectiveness in undiscounted incremental life years (LYs) and undiscounted incremental quality-adjusted life years (QALYs) compared to standard care (intensified surveillance for breast cancer). incr.: incremental; LYs: Life years; PBM: Prophylactic bilateral mastectomy; PBSO: Prophylactic bilateral salpingo-oophorectomy; QALYs: quality-adjusted life years; y: years of age

Compared to standard of care, PBM alone at age 30 yielded 2.8 LYG (4.9 QALYs) gained and PBSO alone at age 30 yielded 2.5 LYG (4.4 QALYs) gained. In contrast, a combined PBM plus PBSO at age 30 yielded 6.3 LYG and was the most effective strategy in terms of life expectancy. PBM at age 30 and delayed PBSO at age 35 yielded 11.1 QALYs and was the most effective strategy in terms of QALYs. Delaying the first surgery and/or delaying the second surgery by more than 5 years resulted in a reduction in life expectancy and QALYs. Detailed results on benefits in terms of life-years and QALYs gained for each evaluated strategy are presented in Fig. 2.

### Cost effectiveness

**Fig. 3:**
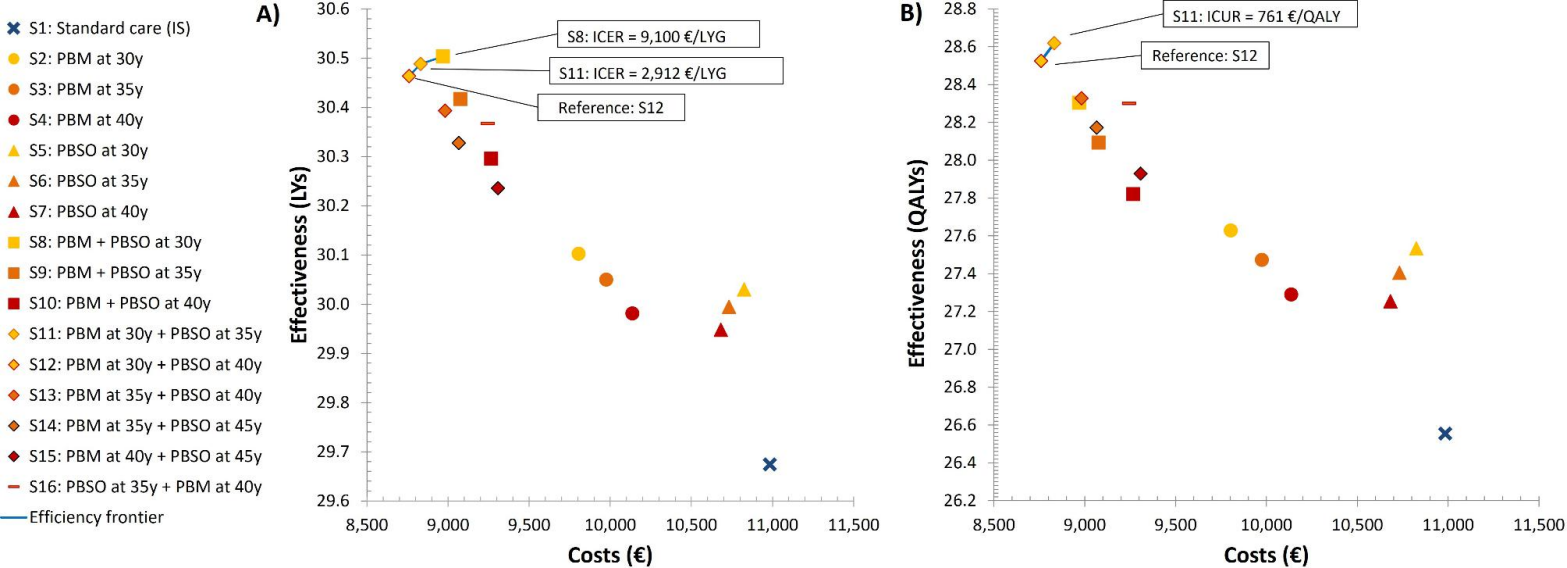
**Cost-effectiveness plane of different preventive strategies:(A)** discounted total life-time costs (in €) versus effectiveness expressed in discounted total life years (LYs) and **(B)** discounted total life-time costs (in €) versus discounted total quality-adjusted life years (QALYs). The respective stepwise ICER (in €/LY) and ICUR (in €/QALY) on the efficiency frontier (blue line) are shown in boxes. Reference strategy: PBM at age 30 and PBSO at age 40 (S12) PBM: Prophylactic bilateral mastectomy; PBSO: Prophylactic bilateral salpingo-oophorectomy; y: years of age

All intervention strategies were more effective and less costly than IS alone. Combined strategies with PBM at age 30 (i.e., PBM at age 30 plus delayed PBSO) dominated all other strategies. A serial combination of PBM at age 30 and delayed PBSO at age 40 was the least costly strategy at total discounted costs of €8,760 and more effective than standard care (intensified surveillance), thus being the reference strategy (Fig. 3A, B). Considering life expectancy only, moving from PBM at age 30 plus PBSO at age 40 to the next more effective strategy PBM at age 30 plus PBSO at age 35 yielded an ICER of 2,912 €/LYG. Moving from PBM at age 30 plus PBSO at age 35 to PBM plus PBSO at age 30 yielded an ICER of 9,100 €/LYG (Fig. 3A). Considering women’s quality of life in terms of QALYs gained, PBM plus PBSO at age 30 was dominated by strategies with delayed PBSO (Fig. 3B). Among the non-dominated strategies, PBM at age 30 plus PBSO at age 40 was the least costly strategy. Moving from this strategy to the next more effective strategy PBM at age 30 plus PBSO at age 35 yielded an ICUR of 761 €/QALY gained (Fig. 3B).

### Sensitivity analyses

In all sensitivity analyses, model results were robust against variations in costs, utilities, and effect measures with a stable rank order of evaluated strategies. Model results were sensitive regarding variations in the annual discount rate only. For a low discount rate (1%) the strategy PBM at age 30 plus a PBSO at age 40 was dominated by PBM at age 30 plus PBSO at age 35. With an annual discount rate of 10% several strategies became undominated with standard care being the new reference strategy. Results of the sensitivity analyses are summarized in additional file 3 (Table S2 A: Summary of sensitivity analysis results: Incremental cost-effectiveness ratios compared to the next non-dominated strategy, Table S2 B: Summary of Sensitivity analysis results: incremental cost – utility ratios compared to the next non-dominated strategy).

## Discussion

Our model-based results suggest that PBM plus PBSO at age 30 is highly effective regarding life years, but serial PBM at age 30 with delayed PBSO between age 35 and 40 is most effective considering QALYs for women with *BRCA-1/2* mutations in Germany. Also, PBM at age 30 followed by delayed PBSO at age 35 or 40 can be considered highly cost effective when applying a recently published model-based WTP threshold for Germany [23]. Thus, our findings support current recommendations of the German guidelines regarding age and type of prophylactic surgery [3, 6].

The reduced costs for strategies with PBM at age 30 can be explained by a reduction in costs for intensified surveillance after surgical removal of the breasts; a MRI is no longer needed in these women. Thus, the earlier a bilateral mastectomy is conducted, the lower are the costs for the respective strategy. Moreover, cancer risk is reduced drastically at an early age, resulting in lower cancer treatment costs for the remaining lifetime. Regarding QALYs, only serial strategies were not dominated. This suggests that when taking into account health-related quality of life, an early PBSO may not be the best option for a woman. For woman’s life an early PBSO implies that women are put into artificial menopause and lose their ability to have children at a relatively young age. Importantly, our decision analyses show that IS for breast cancer alone was the most costly and the least effective strategy. Naturally, this “strategy” includes women who have not yet decided for or against prophylactic surgery. In order to prevent cancer effectively and in time, a serial prophylactic surgery should be offered and discussed in a patient-shared decision-making setting as an option to such women along with explicitly communicating the potential losses associated with deferring the decision.

While there are some studies that have evaluated the cost-effectiveness of genetic testing, screening and/or prevention in women with elevated risk for breast and/or ovarian cancer, for example, Ashkenazi Jewish women [32] and women with *BCRA-1/2* mutation [33, 34] (for a recent review see Sroczynski et al. 2020 [35]), there are currently, only two other studies that have evaluated the cost-effectiveness of different prevention strategies in *BRCA-1/2* mutation carriers for the German health care context [21]. While Müller et al. 2019 [36] focus on the cost-effectiveness of genetic testing for identifying *BRCA-1/2* mutation carriers followed by different prevention strategies compared to no genetic testing, Müller et al. 2018 [21] evaluate similar strategies as our study. In contrast to Müller et al. 2018 [21], our results suggest a serial strategy of PBM at 30 years followed by delayed PBSO to be cost effective, whereas in Müller et al. 2018 [21] this strategy was dominated by a non-serial combination of PBM and PBSO at age 30. The strength of our analysis is that we included a wider age range of serial combinations of PBM and PBSO. A consideration of a wider range of serial combinations is crucial as it firstly reflects the current recommendations of the German guidelines and secondly and most importantly the findings of this study show that a delayed PBSO not only suggests having a positive impact on the quality of life of women but also to be a cost-effective option for women. In contrast to Müller et al. 2018 [21], our model considers different cancer stages in more detail and therefore accounts for differences in stage-specific survival rates, utilities, and costs. In addition, modeling undetected and detected cancer and calibrating to the reported age- and stage-specific incidences, allows for detailed clinical analysis including risk and mortality reduction.

Clearly, a decision for undergoing prophylactic surgery may have a huge impact on the woman’s quality of life. This is reflected in the results of our decision analysis. As suggested by our results, the time point of the second prophylactic surgery might have an influence on the quality of life of the remaining lifetime of women. In general, however, a decision on whether to undergo surgery or not in the first place depends on a woman’s individual characteristics such as her familial and personal situation, whether her family planning is completed as well as her individual utilities, level of anxiety and risk attitude. Both prophylactic surgery and IS have positive and negative short- and long-term consequences for women. In our model, we implemented short-term consequences of the surgery itself by quality-of-life reduction (disutility) due to surgery and assumed women to recover from surgery in the following years. Potential long-lasting consequences are not implemented in our model, as their time scale and intensity are unknown. The strategy IS is less invasive than prophylactic surgery, but the least effective option based on our results.

From a health care or decision maker’s perspective, a new medical intervention should have an additional benefit compared to the current standard; with an acceptable benefit-harm relation and cost-effectiveness relation. According to the results of our decision-analytic modeling study, the best option for *BRCA-1/2* mutation carriers is a PBM at the age of 30 followed by delayed PBSO between age 35 and 40, as this is both effective with an acceptable benefit-harm relation considering QALYs and cost effective compared to other strategies.

Strengths of our study include the fact that we developed and applied a validated decision-analytic model, which systematically synthesizes current evidence and state of knowledge of breast and ovarian cancer prevention in German women with *BRCA-1/2* mutations. Compared to existing models, we included a wider range of serial strategies, explicitly modelled cancer stage, and distinguished detected from undetected cancer. We validated the model against observed epidemiological data from German cancer registries to make it applicable to the German health care context. Finally, our model is flexible and can be adapted and used to answer future research questions of similar kind.

As all modeling studies, our study rests on assumption and has several limitations. The vast majority of the limitations are due to the lack of available data. First, the decision model does not consider heterogeneity of the population with respect to different *BRCA* mutation types, as *BRCA*-mutation type specific epidemiological data were not available for all required parts of the model. Literature reports for women with an inherited *BRCA1* mutation a lifetime risk for breast cancer of 65–80% and 37–62% lifetime risk for developing ovarian cancer, while it reports for *BRCA2* mutation carriers a lifetime risk of 45–85% for breast cancer and 11–23% for ovarian cancer [37]. Second, we assumed that stage-specific survival rates do not differ between mutation carriers and non-carriers, as stage-specific treatment procedures are the same. However, there is some evidence that cancer biology is different in *BRCA1* carriers compared to non-carriers, with *BCRA1* carriers having lower [38] or higher [39] and *BRCA2* carriers having higher [40] survival rates, which would result in an over- or under-estimation of the ICER, respectively. But evidence on this topic is still very scarce. Stage-specific survival rates for breast and ovarian cancer in mutation carriers would be necessary to populate the model. Third, we assumed stage distributions for breast and ovarian cancer to be the same as in non-mutation carriers. However, mutation carriers develop cancer at an earlier age [2] compared to non-carriers. Whether this also affects the stage distribution is unknown. Fourth, since information on the proportion, frequency, and costs of stage-specific treatment options of breast and ovarian cancer in Germany is scarce, we assumed breast cancer in stage 1–3 to be treated similarly at similar costs and distinguished these from metastatic breast cancer only. Due to the lack of detailed costs data for ovarian cancer management we distinguished non-advanced from advanced ovarian cancer only. Although treatment resources likely differ between different cancer stages in sensitivity analyses results were shown to be robust. Fifth, we used utility estimates that are based on two different methods (standard gamble [SG] and time trade-off [TTO]) because not all utility estimates were available based on TTO only. Sixth, as deterministic sensitivity analyses show robust results over a wide range of relevant clinical and economic model parameter variation, we refrained from conducting a probabilistic sensitivity analysis. In addition, particularly in this field, there is a lack of evidence on the joint distribution of many of the dependent model parameters. Seventh, in most of the serial strategies, we implemented PBM to be followed by PBSO (in only one strategy PBSO is followed by delayed PBM). This is because the breast cancer risk is higher than the ovarian cancer risk, and therefore, PBM can also provide a greater benefit. Also, a delayed PBSO is reasonable because women undergoing PBSO are put into artificial menopause. This has a major additional impact on women’s quality of life in general, with earlier surgery impacting a women quality of life even more severely. Eighth, although women’s preferences, for instance regarding family planning, are expected to play an important role, they are not considered explicitly in this model. Future studies should integrate women’s preferences in comprehensive patient-shared decision making.

## Conclusion

In conclusion, based on the results from our decision analysis, a combination of prophylactic surgeries is an effective and cost-effective cancer prevention option from a German health care perspective. Prophylactic surgery drastically reduces cancer risk but is also associated with more harms due to short-term invasive surgery complications and long-term unintended psychological effects in women. A delayed PBSO after a PBM may improve women’s quality of life and be a cost-effective prevention strategy. We suggest that efforts should be directed to inform women carefully and thoroughly with *BRCA-1/2* mutations about their options to prevent breast and ovarian cancer. Our findings could potentially be used as an orientation for clinical experts and decision makers in Germany to guide further improvements in the management strategies for *BRCA-1/2* mutation carriers and breast and/or ovarian cancer prevention.

## Electronic supplementary material

Below is the link to the electronic supplementary material.

Additional file 1

Additional file 2

Additional file 3

## Data Availability

All data used or analyzed during this study are included in this published article [and its supplementary information files].

## List of abbreviations

IS: Intensified surveillance
DESTATIS: German federal statistical office
GDP: Gross Domestic Product
ICER: Incremental cost-effectiveness ratio
ICUR: Incremental cost-utility ratio
LY: Life years
LYG: Life-year gained
MCR: Munich Cancer Registry
MRI: Magnet resonance imaging
PBM: Prophylactic bilateral mastectomy
PBSO: Prophylactic bilateral oophorectomy
QALY: Quality-adjusted life year
RKI: Robert Koch Institute
SG: Standard gamble
TTO: Time trade-off
WHO: World Health Organization
WTP: Willingness-to-pay threshold
