# Supplementary material for: Decision-analytic evaluation of the comparative effectiveness and cost-effectiveness of strategies to prevent breast and ovarian cancer in German women with BRCA-1/2 mutations
Source: BMC Cancer. 2023 Jun 26;23:590. doi: 10.1186/s12885-023-10956-6 (PMC10294312; doi:10.1186/s12885-023-10956-6)
Supplement: Supplementary file 1 — Additional file 1 [file 12885_2023_10956_MOESM1_ESM.docx]

**Additional file 1**

***Model calibration***

The model was calibrated in a systematic and hierarchical fashion to fit *BRCA-1/2* mutation carrier specific incidences and stage distribution of breast and ovarian cancer observed in the female population of Germany. We calibrated for each cancer type separately age-specific incidences followed by a calibration of cancer stage distributions. Data from a published meta-analysis (1) were used to calibrate the model to fit *BRCA-1/2*- and age-specific incidences for breast and ovarian cancer (Table S1). The combined incidence estimate for both types of *BRCA* mutations is reported to be based on a proportion of 56.3 % *BRCA-1* and 43.7% *BRCA-2* mutation carriers (1). Epidemiological data from the Munich Cancer Registry (MCR) were used to calibrate the model to fit stage distribution according to pTNM for breast cancer (pT1: 59.2%; pT2: 33.4%; pT3: 4.2 %; pT4: 3.1 %) and FIGO classification for ovarian cancer (FIGO I: 19.7%; FIGO II: 6.6%; FIGO III: 50.7 %; FIGO IV: 23.1 %), respectively. We assumed that *BRCA-1/2* mutation carriers diagnosed with breast cancer have the same stage distribution as non-carriers. We started the calibration assuming annual stage-specific progression probabilities of 0.205 (pT1 to pT2; pT2 to pT3) and 0.343 (pT3 to pT4) for breast cancer. These estimates are based on sojourn time following a model by Rojnik et al. (2). Annual progression probabilities for ovarian cancer were assumed to be 0.7, 0.9 and 0.95 for the progression from FIGO I to II, FIGO II to III and FIGO III to IV, respectively. Progression probabilities from undetected to detected cancer were assumed to take values of 0.25, 0.5, 0.75 and 1 (pT1-4) for breast cancer. For ovarian cancer we assumed 0.13, 0.13, 0.6 and 0.85 (FIGO I-IV). These estimates are based on an autopsy study (3). We assumed ovarian cancer detection probabilities to be lower than for breast cancer as there is no intensified surveillance for ovarian cancer, thus it is less likely to be detected. During calibration, stage-specific progression probabilities were altered until the model predicted observed cancer stage distributions, while holding progression from undetected to detected cancer constant.

After calibration the model predicted *BRCA-1/2*- and age-specific incidences for breast and ovarian cancer matched the observed incidences (1), with an age peak around 49 years for both cancer types. Similarly, model predicted breast and ovarian cancer stage distributions matched observed data provided by the MCR, with 93% of breast cancer cases being distributed to early stages (pT1 and pT2) and 71% of ovarian cancer cases being distributed to late stages (FIGO III and FIGO IV).

For the calibration procedure of incidences and stage distributions of both cancers we used *BRCA-*specific incidences and stage distributions of the normal population, as there were no data on *BRCA*-specific stage distributions available. In order to avoid possible bias, we performed the calibration in a hierarchical way by starting with calibration of cancer incidences, to fit *BRCA*-specific cancer development, followed by the calibration of the stage distributions.

**Table S1. Annual age-specific incidence rates for breast and ovarian cancer in *BRCA-1/2* mutation carriers.** The values were used for calibration of progression from health state ‘Well’ to ‘undetected cancer’. A proportion of 56.3% *BRCA-1* and 43.7% *BRCA-2* mutation carriers was assumed (1).

|  | **Breast cancer** | **Ovarian cancer** | |
| --- | --- | --- | --- |
| **Age group** | *Annual incidence rate* | *Annual incidence rate* |  |
| 20-24 | 0.0002 | 0.00001 | |
| 25-29 | 0.0011 | 0.00002 | |
| 30-34 | 0.0057 | 0.0010 | |
| 35-39 | 0.0123 | 0.0016 | |
| 40-44 | 0.0204 | 0.0052 | |
| 45-49 | 0.0299 | 0.0089 | |
| 50-54 | 0.0226 | 0.0080 | |
| 55-59 | 0.0256 | 0.0099 | |
| 60-64 | 0.0246 | 0.0143 | |
| 65-69 | 0.0271 | 0.0158 | |

**References**

1. Antoniou A, Pharoah PD, Narod S, Risch HA, Eyfjord JE, Hopper JL, et al. Average risks of breast and ovarian cancer associated with BRCA1 or BRCA2 mutations detected in case Series unselected for family history: a combined analysis of 22 studies. American journal of human genetics. 2003;72(5):1117-30.

2. Rojnik K, Naversnik K, Mateovic-Rojnik T, Primiczakelj M. Probabilistic cost-effectiveness modeling of different breast cancer screening policies in Slovenia. Value Health. 2008;11(2):139-48.

3. Guth U, Arndt V, Stadlmann S, Huang DJ, Singer G. Epidemiology in ovarian carcinoma: Lessons from autopsy. Gynecologic oncology. 2015;138(2):417-20.
