# Supplementary material for: Decision-analytic evaluation of the comparative effectiveness and cost-effectiveness of strategies to prevent breast and ovarian cancer in German women with BRCA-1/2 mutations
Source: BMC Cancer. 2023 Jun 26;23:590. doi: 10.1186/s12885-023-10956-6 (PMC10294312; doi:10.1186/s12885-023-10956-6)
Supplement: Supplementary file 2 — Additional file 2 [file 12885_2023_10956_MOESM2_ESM.docx]

**Additional file 2**

***Utility of undetected cancer***

We approximated utility values for undetected cancer via utility values for patients without depression or depression-like symptoms. This is reasonable, because the knowledge of the disease itself and cancer treatment in diagnosed cancer patients has been shown to result in psychological symptoms of distress, such as depression and anxiety in 30-40 % of cancer patients, which is reflected in their health state utility (1-4). Compared with patients with none or minimal level of depression, the health state of cancer patients with depression has a mean absolute decrement of 0.14 in its health utility value (5); this corresponds to a 16% relative decrease in utility due to depression. The utility values of the health states for diagnosed cancer patients include approximately 35% depressed patients with decreased utility. Thus, utility values for non-depressed (i.e., not diagnosed with cancer) patients should be higher. The following approximation (Equation 1) was used to estimate utility of such undiagnosed cancer patients:

Utility_undetected_ = Utility_diagnosed_ + ((Utility_diagnosed_*(16/100))* 0.35) (Equation 1)

**References**

1. Fann JR, Thomas-Rich AM, Katon WJ, Cowley D, Pepping M, McGregor BA, et al. Major depression after breast cancer: a review of epidemiology and treatment. General hospital psychiatry. 2008;30(2):112-26.

2. Quinten C, Coens C, Mauer M, Comte S, Sprangers MA, Cleeland C, et al. Baseline quality of life as a prognostic indicator of survival: a meta-analysis of individual patient data from EORTC clinical trials. The Lancet Oncology. 2009;10(9):865-71.

3. Stark D, Kiely M, Smith A, Velikova G, House A, Selby P. Anxiety disorders in cancer patients: their nature, associations, and relation to quality of life. Journal of clinical oncology : official journal of the American Society of Clinical Oncology. 2002;20(14):3137-48.

4. Brown PO, Palmer C. The preclinical natural history of serous ovarian cancer: defining the target for early detection. PLoS Med. 2009;6(7):e1000114.

5. Fujisawa D, Inoguchi H, Shimoda H, Yoshiuchi K, Inoue S, Ogawa A, et al. Impact of depression on health utility value in cancer patients. Psycho-oncology. 2016;25(5):491-5.
