# Supplementary material for: Decision-analytic evaluation of the comparative effectiveness and cost-effectiveness of strategies to prevent breast and ovarian cancer in German women with BRCA-1/2 mutations
Source: BMC Cancer. 2023 Jun 26;23:590. doi: 10.1186/s12885-023-10956-6 (PMC10294312; doi:10.1186/s12885-023-10956-6)
Supplement: Supplementary file 3 — Additional file 3 [file 12885_2023_10956_MOESM3_ESM.docx]

**Additional file 3**

**Table S2 A) Summary of sensitivity analysis results: incremental cost – effectiveness ratios (ICER; in 2022 Euro/LYG) compared to the next non-dominated strategy**

|  | **ICER (2022 Euro/LYG)*** | | | | | | | | | |
| --- | --- | --- | --- | --- | --- | --- | --- | --- | --- | --- |
| **Strategy** | **Base case** | **Varied parameter** | | | | | | | | |
|  |  | **Costs** | | | **Discount rate** | | | **Effect measures** | | |
|  |  | **50%** | **150%** | **200%** | **1%** | **5%** | **10%** | **50%** | **150%** | **200%** |
| *S12: PBM at 30 + PBSO at 40* | Ref. | Ref. | Ref. | Ref. | Dom. | 2,285 | 49,694 | Ref. | Ref. | Ref. |
| *S11: PBM at 30 + PBSO at 35* | 2,912 | 1,642 | 3,812 | 4,341 | Ref. | 10,527 | 61,707 | 2,963 | 2,876 | 2,853 |
| *S8: PBM+PBSO at 30* | 9,100 | 4,749 | 13,053 | 16,607 | 1,022 | 25,952 | 134,483 | 9,517 | 8,741 | 8,429 |
| *S13: PBM at 35 + PBSO at 40* | Dom. | Dom. | Dom. | Dom. | Dom. | 161 | 47,615 | Dom. | Dom. | Dom. |
| *S14: PBM at 35 + PBSO at 45* | Dom. | Dom. | Dom. | Dom. | Dom. | Dom. | Dom. | Dom. | Dom. | Dom. |
| *S9: PBM+PBSO at 35* | Dom. | Dom. | Dom. | Dom. | Dom. | Dom. | Ext.Dom | Dom. | Dom. | Dom. |
| *S16: PBSO at 35 + PBM at 40* | Dom. | Dom. | Dom. | Dom. | Dom. | Dom. | 16,137 | Dom. | Dom. | Dom. |
| *S10: PBM+PBSO at 40* | Dom. | Dom. | Dom. | Dom. | Dom. | Dom. | 12,873 | Dom. | Dom. | Dom. |
| *S15: PBM at 40 + PBSO at 45* | Dom. | Dom. | Dom. | Dom. | Dom. | Ref. | 12,586 | Dom. | Dom. | Dom. |
| *S2: PBM at 30* | Dom. | Dom. | Dom. | Dom. | Dom. | Dom. | Dom. | Dom. | Dom. | Dom. |
| *S3: PBM at 35* | Dom. | Dom. | Dom. | Dom. | Dom. | Dom. | Dom. | Dom. | Dom. | Dom. |
| *S4: PBM at 40* | Dom. | Dom. | Dom. | Dom. | Dom. | Dom. | Dom. | Dom. | Dom. | Dom. |
| *S7: PBSO at 40* | Dom. | Dom. | Dom. | Dom. | Dom. | Dom. | 182 | Dom. | Dom. | Dom. |
| *S6: PBSO at 35* | Dom. | Dom. | Dom. | Dom. | Dom. | Dom. | Ext.Dom | Dom. | Dom. | Dom. |
| *S5: PBSO at 30* | Dom. | Dom. | Dom. | Dom. | Dom. | Dom. | Dom. | Dom. | Dom. | Dom. |
| *S1: Standard care* | Dom. | Dom. | Dom. | Dom. | Dom. | Dom. | Ref. | Dom. | Dom. | Dom. |

Dom.: dominated; Ext.: extended; ICER: incremental cost-effectiveness ratio; LYG: Life years gained; PBM: Prophylactic bilateral mastectomy; PBSO: Prophylactic bilateral salpingo-oophorectomy; Ref: reference strategy *rounded values

**Table S2 B) Summary of Sensitivity analysis results: incremental cost – utility ratios (ICUR; in 2022 Euro/QALY) compared to the next non-dominated strategy**

|  | **ICUR (2022 Euro/QALY)*** | | | | | | | | | | | | | |
| --- | --- | --- | --- | --- | --- | --- | --- | --- | --- | --- | --- | --- | --- | --- |
| **Strategy** | **Base case** | **Varied parameter**  **Costs** | | | **Varied parameter**  **Discount rate** | | | **Varied parameter**  **Effect measures** | | | **Varied parameter**  **Utility values** | | | |
|  |  | **50%** | **150%** | **200%** | **1%** | **5%** | **10%** | **50%** | **150%** | **200%** | **80%** | **90%** | **110%** | **120%** |
| *S12: PBM at 30 + PBSO at 40* | Ref. | Ref. | Ref. | Ref. | Dom. | 534 | Dom. | Ref. | Ref. | Ref. | Ref. | Ref. | Ref. | Ref. |
| *S11: PBM at 30 + PBSO at 35* | 761 | 429 | 996 | 1,135 | Ref. | 1,509 | 4,246 | 770 | 755 | 752 | 838 | 798 | 728 | 697 |
| *S8: PBM+PBSO at 30* | Dom. | Dom. | Dom. | Dom. | Dom. | Dom. | Dom. | Dom. | Dom. | Dom. | Dom. | Dom. | Dom. | Dom. |
| *S13: PBM at 35 + PBSO at 40* | Dom. | Dom. | Dom. | Dom. | Dom. | 45 | Dom. | Dom. | Dom. | Dom. | Dom. | Dom. | Dom. | Dom. |
| *S14: PBM at 35 + PBSO at 45* | Dom. | Dom. | Dom. | Dom. | Dom. | Dom. | Dom. | Dom. | Dom. | Dom. | Dom. | Dom. | Dom. | Dom. |
| *S9: PBM+PBSO at 35* | Dom. | Dom. | Dom. | Dom. | Dom. | Dom. | Dom. | Dom. | Dom. | Dom. | Dom. | Dom. | Dom. | Dom. |
| *S16: PBSO at 35 + PBM at 40* | Dom. | Dom. | Dom. | Dom. | Dom. | Dom. | 1,910 | Dom. | Dom. | Dom. | Dom. | Dom. | Dom. | Dom. |
| *S10: PBM+PBSO at 40* | Dom. | Dom. | Dom. | Dom. | Dom. | Dom. | Dom. | Dom. | Dom. | Dom. | Dom. | Dom. | Dom. | Dom. |
| *S15: PBM at 40 + PBSO at 45* | Dom. | Dom. | Dom. | Dom. | Dom. | Ref. | Dom. | Dom. | Dom. | Dom. | Dom. | Dom. | Dom. | Dom. |
| *S2: PBM at 30* | Dom. | Dom. | Dom. | Dom. | Dom. | Dom. | Dom. | Dom. | Dom. | Dom. | Dom. | Dom. | Dom. | Dom. |
| *S3: PBM at 35* | Dom. | Dom. | Dom. | Dom. | Dom. | Dom. | Dom. | Dom. | Dom. | Dom. | Dom. | Dom. | Dom. | Dom. |
| *S4: PBM at 40* | Dom. | Dom. | Dom. | Dom. | Dom. | Dom. | Dom. | Dom. | Dom. | Dom. | Dom. | Dom. | Dom. | Dom. |
| *S7: PBSO at 40* | Dom. | Dom. | Dom. | Dom. | Dom. | Dom. | 28 | Dom. | Dom. | Dom. | Dom. | Dom. | Dom. | Dom. |
| *S6: PBSO at 35* | Dom. | Dom. | Dom. | Dom. | Dom. | Dom. | 1,363 | Dom. | Dom. | Dom. | Dom. | Dom. | Dom. | Dom. |
| *S5: PBSO at 30* | Dom. | Dom. | Dom. | Dom. | Dom. | Dom. | Dom. | Dom. | Dom. | Dom. | Dom. | Dom. | Dom. | Dom. |
| *S1: Standard care* | Dom. | Dom. | Dom. | Dom. | Dom. | Dom. | Ref. | Dom. | Dom. | Dom. | Dom. | Dom. | Dom. | Dom. |

Dom.: dominated; ICUR: incremental cost-utility ratio; LYG: Life years gained; PBM: Prophylactic bilateral mastectomy; PBSO: Prophylactic bilateral salpingo-oophorectomy; QAYL: quality-adjusted life year; Ref: reference strategy *rounded values
